# Self‐Included Fluorescent Probes via Azocalix[4]arene–Dye Conjugation for Activatable and Stable NIR Imaging

**DOI:** 10.1002/advs.78729

**Published:** 2026-11-09

**Authors:** Shun‐Yu Yao, Ze‐Han Wang, Chenyin He, Lidian Zheng, An‐Kang Ying, Kang Cai, Ai‐Feng Liu, Xiufeng Xiao, Dong‐Sheng Guo

**Affiliations:** ^1^ College of Chemistry and Materials Science Fujian Provincial Key Laboratory of Advanced Materials Oriented Chemical Engineering Fujian Normal University Fuzhou China; ^2^ Orthopedics Department National Clinical Research Center for Chinese Medicine Acupuncture and Moxibustion First Teaching Hospital of Tianjin University of Traditional Chinese Medicine Tianjin China; ^3^ College of Chemistry State Key Laboratory of Medicinal Chemical Biology Frontiers Science Center For New Organic Matter Key Laboratory of Functional Polymer Materials (Ministry of Education) Collaborative Innovation Center of Chemical Science and Engineering (Tianjin) Nankai University Tianjin China

**Keywords:** azocalixarene, fluorescent probes, hypoxia‐responsive, precise activation, self‐inclusion

## Abstract

Near‐infrared (NIR) fluorescent probes are crucial tools in bioimaging; however, the challenge of improving their stability and targeting precision remains a key issue. In this work, we present a novel strategy for designing Self‐Included Fluorescent Probes (SIFPs) using a covalent macrocyclic host‐dye conjugate approach. By covalently linking NIR dyes, such as indole heptamethine cyanine fluorescent probes, to azocalix[4]arene carriers, we leverage the unique properties of the macrocyclic host to encapsulate and protect the dyes intramolecularly, while enhancing their stability and preventing off‐target leakage. Importantly, the azocalixarene's hypoxia‐responsive azo linkers enable targeted activation of the dye in tumor environments, improving imaging accuracy. Through comprehensive in vitro and in vivo experiments, we demonstrate that SIFPs significantly outperform conventional probes and their host‐guest binary complexes in terms of stability and imaging precision. This strategy not only combines the benefits of covalent and non‐covalent binding but also offers an effective method for enhancing the design of activatable, stable fluorescent probes.

## Graphical abstract

Self‐Included Fluorescent Probes (SIFPs), composed of self‐included macrocyclic carrier‐dye conjugates, encapsulate and protect covalently attached dyes, preventing leakage and enhancing stability. Azocalixarene‐based hypoxia activation enables precise tumor‐specific imaging. This strategy merges covalent and non‐covalent advantages, offering a versatile method to convert existing dyes into activatable, stable probes.

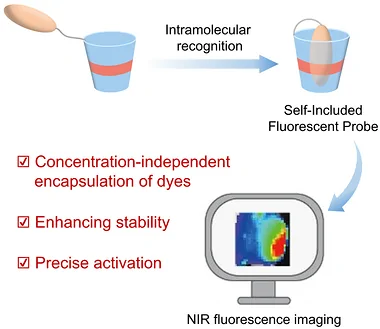

## 1 Introduction

Fluorescence imaging using organic dyes as probes has become an indispensable tool in the detection and imaging of endogenous species as well as imaging‐guided diagnosis and therapy [1, 2, 3, 4, 5, 6]. However, probes with an “always‐on” signal emit fluorescence in both normal tissues and lesion sites, severely compromising imaging precision [7, 8, 9, 10]. In contrast, activatable probes can be selectively “turned on” at the target site by specific biomarkers, providing a higher tumor‐to‐normal tissue ratio and more precise imaging [11, 12, 13, 14, 15, 16]. Particularly, near‐infrared (NIR) probes offer reduced background autofluorescence, lower tissue damage, and enhanced tissue penetration and resolution [17, 18, 19, 20]. Nevertheless, NIR dye probes often suffer from limited chemical and/or photostability [21, 22, 23, 24, 25]. Currently, most studies focus on covalent modifications at specific positions on the fluorophore to introduce stimulus‐responsiveness and improve stability [26, 27]. However, such localized modifications can drastically alter the physicochemical properties and biotoxicity of the dye, and full protection of the whole dye molecule remains challenging [28, 29, 30]. This limits the general applicability and effectiveness of these approaches in stabilizing the probe. Thus, developing a versatile molecular design strategy that imparts both activatable functionality and excellent stability to fluorescence probes is of significant research importance.

In addition to covalent modifications, non‐covalent molecular recognition or assembly can also be employed to modulate the properties of fluorescent dyes. For example, supramolecular macrocycles like cyclodextrins [31, 32, 33] or cucurbiturils [34, 35, 36] can alter the luminescent properties of dyes through encapsulation within their confined cavities, effectively protecting the dye molecules and significantly enhancing their chemical and photostability. Besides, the reversibility of non‐covalent interactions can provide stimulus‐responsive features. Moreover, many of these macrocyclic hosts can encapsulate a variety of different dyes, offering versatility [37, 38, 39, 40]. Thus, designing activatable fluorescence probes based on a host‐guest strategy has notable advantages. However, non‐covalent interactions are inherently reversible and concentration‐dependent, meaning that dilution during in vivo circulation and the presence of complex interfering substances in biofluids can weaken host‐guest binding, leading to off‐target leakage and reduced imaging precision [41, 42, 43, 44]. Therefore, there is a pressing need to develop innovative molecular design strategies that maintain the advantages of host‐guest interactions while overcoming their concentration‐dependence limitations.

To address this, we introduce the concept of a Self‐Included Fluorescent Probe (SIFPs), where a dye probe is covalently attached to a macrocyclic host. In this design, the probe and host form an intramolecular self‐inclusion complex, allowing the macrocycle's confined cavity to protect the dye molecule and modulate its photophysical properties. Importantly, self‐inclusion complexes are inherently non‐concentration dependent, preventing off‐target leakage during in vivo circulation [45, 46]. Moreover, if the macrocycle or covalent linker can be cleaved under specific stimuli, this design enables activatable fluorescence. For our SIFPs design, we chose an azocalix[4]arene derivative as the macrocyclic host, because: i) it acts as a “super quencher,” efficiently quenching the fluorescence of the dye through host‐guest interaction [47, 48, 49, 50]; ii) the azo groups in azocalix[4]arene undergo cleavage under hypoxic conditions, releasing the encapsulated dye [51, 52, 53, 54]. These dual characteristics make azocalix[4]arene a perfect macrocyclic host for implementing the SIFP strategy (Scheme 1). Dye molecules that can be covalently modified are theoretically suitable for this design. In this work, we selected two indole heptamethine cyanine dyes, ICG and IR780, both widely used in biomedical imaging research but prone to stability issues [55, 56, 57]. Using the azocalix[4]arene‐ICG/IR host‐dye conjugates, we demonstrated through both in vitro and in vivo experiments that the SIFP strategy effectively imparts activatable functionality and significantly enhances the stability of NIR dyes, making it a promising approach for designing activatable NIR probes.

**SCHEME 1:**
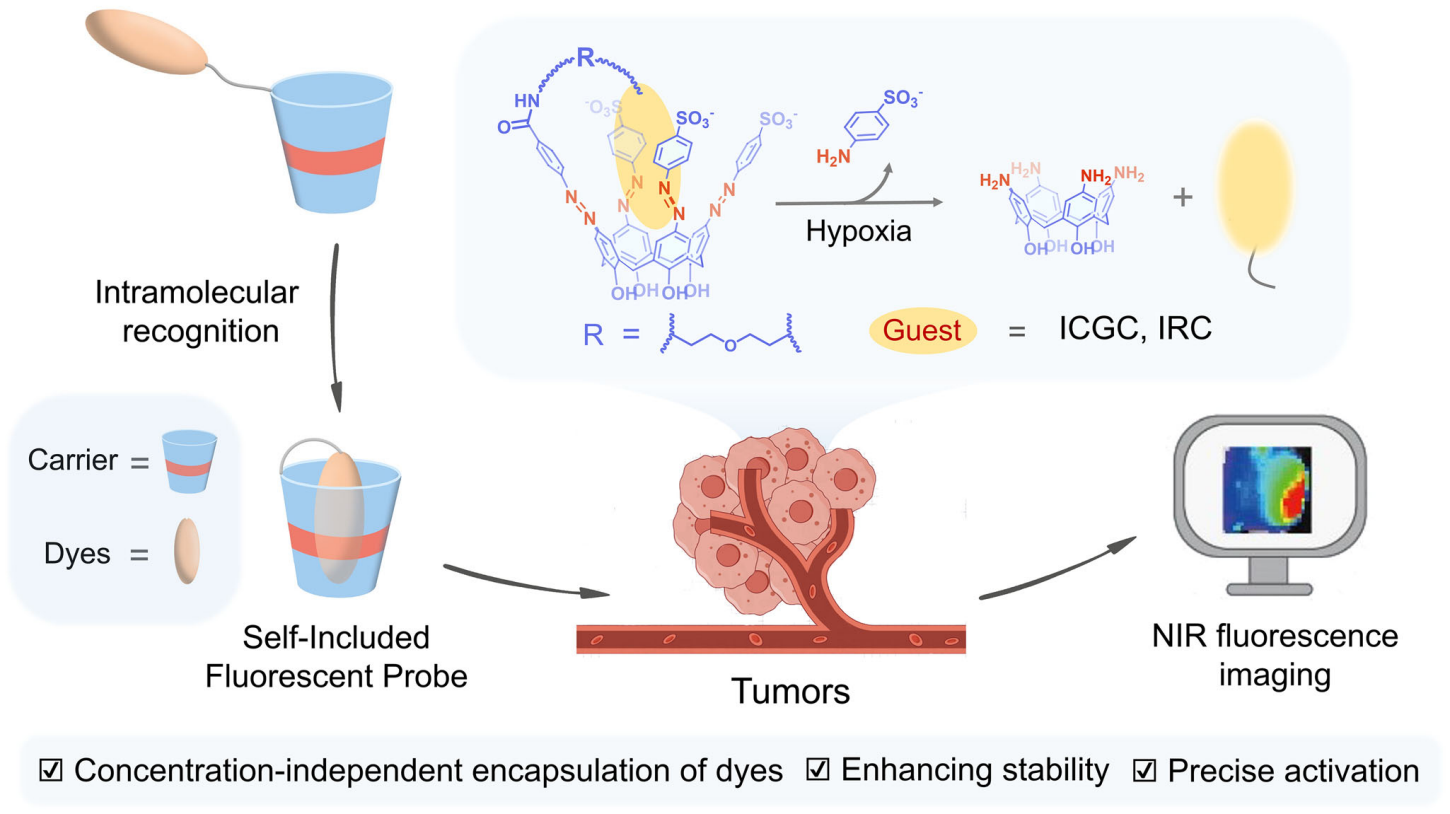
The design of the cleavable SIFPs based on host‐dye conjugates using azocalix[4]arene as the host, and a schematic illustration of the hypoxia‐triggered activation of fluorescent dyes.

## 2 Results and Discussion

### 2.1 The Design, Syntheses and Characterization of the Two Azocalixarene‐Dye Conjugates

The formation of self‐inclusion host‐guest conjugates occurs when a guest molecule is covalently attached to a macrocyclic host. Previous studies have demonstrated that, when the linker length is optimized, intramolecular self‐inclusion predominates over intermolecular polymerization, resulting in enhanced stability even under highly diluted conditions [58, 59, 60, 61]. In this context, we selected a linker composed of two ethylene glycol units to connect the macrocyclic carrier, C4A, and the guest dyes. Two indole heptamethine cyanine dyes, ICG‐COOH (ICGC) and IR‐COOH (IRC) were chosen as guests, leading to the formation of two carrier‐dye conjugates: ICG‐C4A and IR‐C4A (Figure 1a).

**FIGURE 1:**
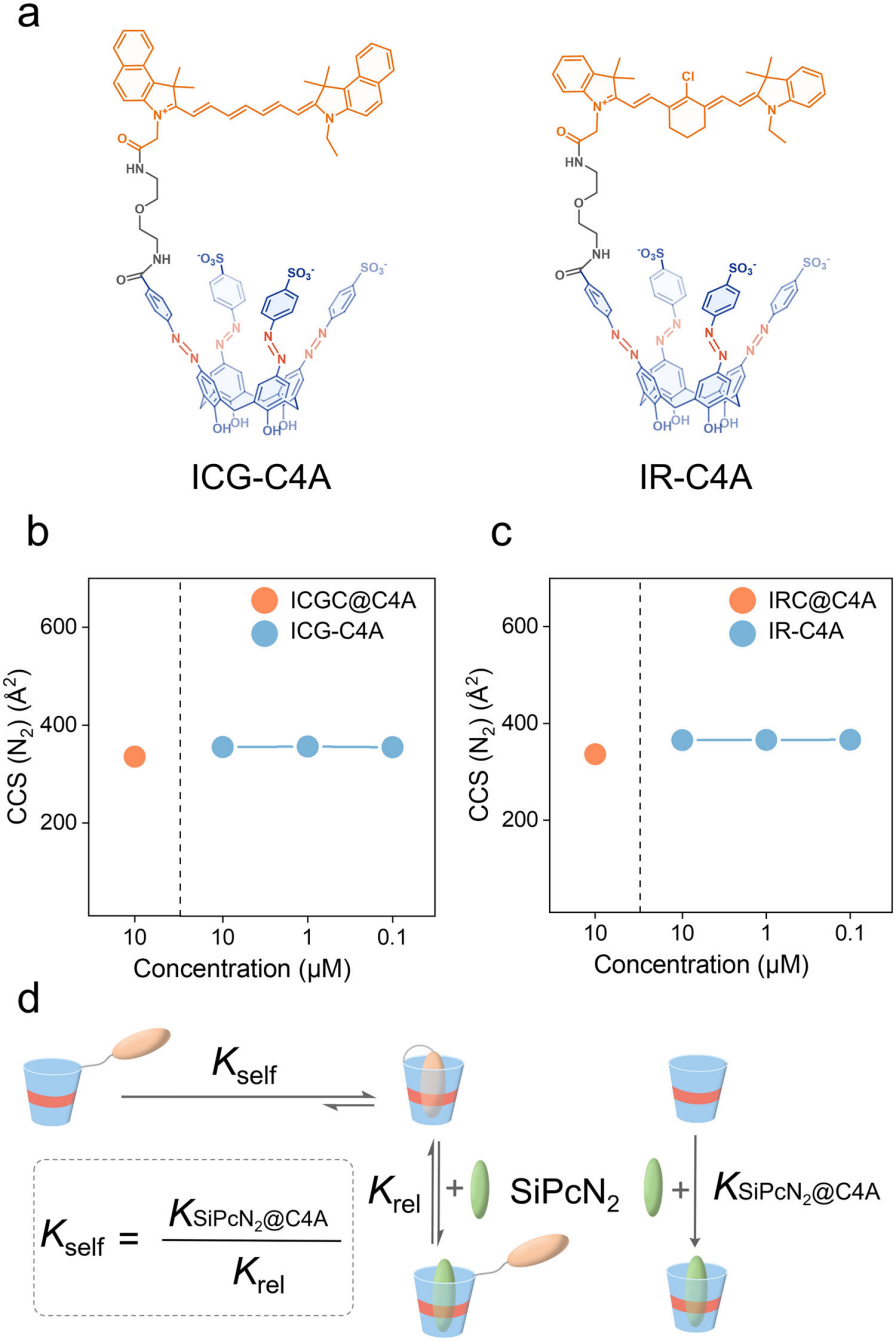
(a) The chemical formulas of the two azocalixarene‐dye conjugates. (b,c) Collision cross section (CCS) of the two azocalixarene‐dye conjugates and their corresponding binary complexes determined by TWIMS–MS. (d) Schematic illustration of the determination method for self‐inclusion binding constants.

These two conjugations were characterized using nuclear magnetic resonance (NMR) spectroscopy and high‐resolution mass spectrometry (HRMS) (Figures S1–S12). Since the self‐included conjugates are prone to macrocycle‐mediated intermolecular assembly at elevated concentrations in aqueous solution, which resulting in severe NMR signal broadening and spectral overlap, neither ^1^H NMR nor 2D diffusion‐ordered spectroscopy (DOSY NMR) is suitable for evaluating the self‐inclusion state. To characterize the self‐inclusion behavior at biologically relevant low concentrations, we instead employed travelling wave ion mobility spectrometry‐mass spectrometry (TWIMS‐MS) to obtain conformational information through collision cross section (CCS) determination of both covalently linked carrier‐dye conjugates and their non‐covalently bound counterparts (Table S1 and Figures S20 and S21). For example, at a concentration of 10.0 µM, the collision cross section (CCS) of ICG‐C4A was determined to be 355.59 Å^2^ (Figure 1b), closely matching that of the ICGC@C4A binary complex (335.72 Å^2^). Upon diluting the ICG‐C4A solution to 1.0 µM and 0.1 µM, the CCS values remained stable at 356.58 Å^2^ and 355.39 Å^2^, respectively, indicating no significant change in the CCS values with decreasing concentration. These results support the formation of self‐inclusion complex, with a molecular size similar to that the ICGC@C4A binary complex. In order to validate the universality of this self‐inclusion strategy, we assessed the CCS (Figure 1c) for IR‐C4A. The experimental results consistently demonstrated that covalently linking different guest molecules to the macrocyclic host leads to the formation of stable self‐inclusion complexes. Furthermore, the UV–vis absorption spectra of ICG‑C4A and IR‑C4A recorded over the concentration range of 0.1 µM to 20.0 µM showed that their absorption maxima remained unchanged without noticeable red‐ or blueshifts, and the absorbance exhibited an excellent linear correlation with concentration (Figure S23e–h). These results suggest that the self‐included fluorescent probes exist predominantly as monomeric intramolecular self‑inclusion complexes in aqueous solution, rather than as significant concentration‑dependent aggregates.

Competitive fluorescence titration using rhodamine B (RhB) as the reporter dye determined the binding constants of ICGC and IRC with C4A to be (1.61 ± 0.45) × 10^7^ M^−1^ and (2.94 ± 0.77) × 10^7^ M^−1^(Figure S22a–f), respectively, indicating effective binding of both dyes to C4A and thus providing a reliable basis for the construction of the self‐included fluorescent probes. Fluorescence titration experiments were performed to determine the binding constants (*K*_self_) of the self‐inclusion complexes (Figure 1d). The binding constant (*K*_a_) of C4A with silicon phthalocyanine (SiPcN_2_) was measured as (4.58 ± 0.43) × 10^7^ M^−1^. Subsequently, the competition binding constants (*K*_rel_) between the two self‐inclusion complexes and SiPcN_2_ were measured (Figure S22g–l). The self‐inclusion binding constants were then derived from *K*_self_ = *K*_a_ / *K*_rel_ [62, 63, 64] (Table 1). The *K*_self_ of ICG‐C4A and IR‐C4A were determined to be 5.76 × 10^2^ and 7.32 × 10^2^, respectively, indicating exceptionally high encapsulation efficiencies over 99% for these inclusion complexes.

**TABLE 1:** Self‐inclusion binding constants of the two azocalixarene‐dye conjugates.

Name	*K*_self_
ICG‐C4A	5.76 × 10^2^
IR‐C4A	7.32 × 10^2^

### 2.2 Stability Evaluation

The stability of the self‐inclusion fluorescent probe (ICG‐C4A) was assessed by comparing it to free ICGC, ICGA (azo group modified ICGC, the structural characterization is presented in Figures S13–S16) and the ICGC@C4A binary complex. The chemical stability of ICGC, ICGA, ICGC@C4A, and ICG‑C4A in phosphate‑buffered saline (PBS) and 10% fetal bovine serum (FBS) was evaluated by monitoring the time‑dependent changes in their UV–vis absorption (Figures S23 and S24). As shown in Figure 2a,b, the absorption of both free ICGC and ICGA dropped to less than 20% of their initial values after 96 h in both PBS and 10% FBS solutions, indicating poor chemical stability of ICGC and ICGA. In contrast ICGC@C4A retained 70% and 57% of its original intensity in PBS and FBS solutions, respectively, indicating enhanced chemical stability due to host encapsulation. The increased dissociation of the host‐guest complex in FBS likely contributed to the higher ICGC decomposition in FBS compared to PBS. Notably, the ICG‐C4A group exhibited 92% of its original absorption intensity in PBS after 96 h, with an only slight decrease to 87% in the FBS medium, highlighting its superior chemical stability. Additionally, the photo‐stability of ICG‐C4A and the control groups (Figure 2c) were examined in PBS solutions. After 8 min of 808 nm laser irradiation, the absorption intensity of ICG in the free ICGC, ICGA, ICGC@C4A and ICG‐C4A groups dropped to 11%, 16%, 75%, and 87% of the initial intensities, indicating superior photostability for the self‐inclusion complex. This enhanced stability may arises from three factors:i) The encapsulation of ICGC within the macrocyclic cavity effectively protects it from external degradative interferents; ii) The intramolecular inclusion motif established via conjugation effectively prevents ICGC from dissociating; iii) The confined cavity restricts molecular motion, suppressing non‐radiative pathways that cause photodegradation.

**FIGURE 2:**
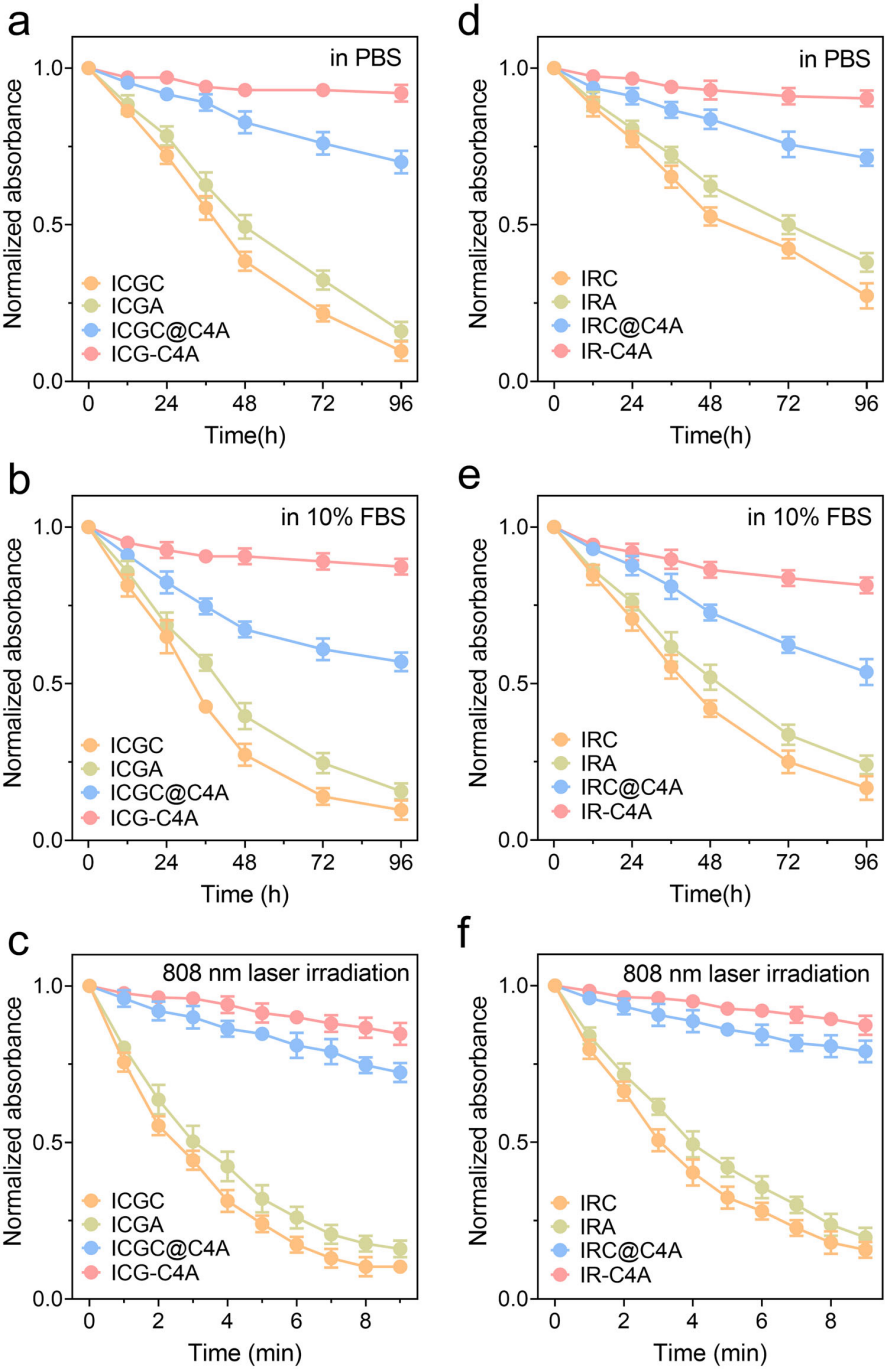
(a) Evaluating the chemical stability of ICGC, ICGA, ICGC@C4A (binary complex) and ICG‐C4A (self‐inclusion complex) in phosphate buffer saline (PBS) solution (pH = 7.4) by tracking the UV–vis absorption at different time periods. (b) Evaluating the chemical stability of ICG‐C4A and the control groups in 10% fetal bovine serum (FBS) solution by tracking the UV–vis absorption at different time periods. (c) Evaluating the photo‐stability of ICG‐C4A and the control groups under laser irradiation in PBS solution (808 nm, 1 W cm^−2^) by tracking the UV–vis absorption intensity at different time periods. (d) Evaluating the chemical stability of IRC, IRA, IR@C4A (binary complex) and IR‐C4A (self‐inclusion complex) in PBS solution (pH = 7.4) by tracking the UV–vis absorption at different time periods. (e) Evaluating the chemical stability of IR‐C4A and the control groups in 10% FBS solution by tracking the UV–vis absorption at different time periods. (f) Evaluating the photo‐stability of IR‐C4A and the control groups under laser irradiation in PBS solution (808 nm, 1 W cm^−2^) by tracking the UV–vis absorption at different time periods. For all stability assays, the absorbance of each compound was recorded at its respective characteristic maximum absorption wavelength: ICGC at 776 nm, ICGA at 735 nm, ICGC@C4A at 735 nm, ICG‐C4A at 724 nm, IRC at 770 nm, IRA at 726 nm, IRC@C4A at 730 nm, and IR‐C4A at 718 nm. All data represent the mean ± s.d. (*n* = 3).

The chemical and photo‐stability of the IR‐C4A were also evaluated using free IRC, IRA (azo group modified IRC, the structural characterization is presented in Figures S17–S19) and IRC@C4A as the control groups (The UV–vis absorption spectrum have shown in Figures S23 and S25). As shown in Figure 2d,e, IR‐C4A retained 90.3% and 81.3% of its original intensity in PBS and FBS solutions, respectively, which were much higher than those of free IR‐780, IRA and IRC@C4A. Moreover, IR‐C4A also exhibited significantly enhanced photostability compared with free IRC, IRA and IRC@C4A (Figure 2f). These findings underscore the significant protective effect of the self‐inclusion strategy, enhancing both chemical and photo‐stability of the encapsulated dyes.

### 2.3 Hypoxia‐Responsive Property Evaluation

Sodium dithionite (SDT) was added to the solutions of ICG‐C4A and IR‐C4A, respectively, and the absorbance at 420 nm was monitored continuously by using UV–vis spectroscopy. Both ICG‐C4A and IR‐C4A displayed broad absorption peaks above 400 nm, attributed to *n*–*π* ^*^ transitions of the azo groups. Upon the addition of excess SDT, a chemical mimic of azo reductase, the absorption of the azo group at 420 nm significantly decreased (Figure 3a,b). Furthermore, the self‐inclusion fluorescent probes were incubated with rat liver microsomes and nicotinamide adenine dinucleotide phosphate (NADPH) under normoxic and hypoxic conditions to evaluate their hypoxia‐responsiveness based on the fluorescence recovery (Figure S26). The fluorescence intensity of the dyes recovered by over 80% within 15 min under hypoxic conditions, whereas almost no fluorescence recovery was detected under normoxic conditions (Figure 3c,d). Since, the binding affinity of dyes to azocalix[4]arene and aminocalix[4]arene, the reduction product, differed significantly, the cleavage of the azo group in azocalix[4]arene led to the dye release from the cavity, resulting in the gradual recovery of fluorescence [65, 66, 67]. Furthermore, aminocalixarene and ICGC derivatives with aromatic amine fragments (ICGC‐NH_2_) were detected by ultra‐performance liquid chromatography‐tandem‐mass spectrometry (UPLC‐MS/MS) in the supernatant of cell lysates derived from cells treated with ICG‑C4A under hypoxic conditions (Figure S27), directly confirming that the azo groups are cleaved under hypoxia. To evaluate the environmental stability and hypoxia‐activation specificity of ICG‐C4A and IR‐C4A, we examined their fluorescence recovery under normoxic and hypoxic conditions in the presence of microsomes, NADPH, nicotinamide adenine dinucleotide (NADH), homocysteine (Hcy), cysteine (Cys), glutathione (GSH) and no additive, along with a positive control containing both microsomes and NADPH (Figure S28). Significant recovery occurred only in the presence of both microsomes and NADPH under hypoxia, confirming the excellent stability and hypoxia‐ specific activation of both probes. Collectively, these results demonstrate that the self‐inclusion fluorescent probe exhibits remarkable hypoxia‐responsive properties, enabling controlled activation of the probe under hypoxic conditions. Moreover, the photophysical properties of the dyes released from ICG‐C4A and IR‐C4A upon hypoxic activation remain essentially unchanged relative to those of ICGC and IRC (Table S2), even though the released dyes are not the native dyes, suggesting that ICG‐C4A and IR‐C4A are suitable for near‐infrared in vivo fluorescence imaging.

**FIGURE 3:**
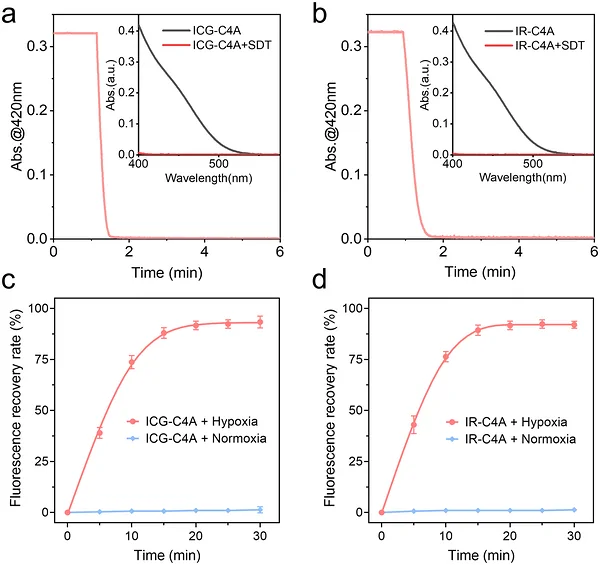
(a) Absorbance at 420 nm of ICG‐C4A (10.0 µM) as a function of time following addition of SDT (1.0 mM). Inset: Absorbance spectra of ICG‐C4A (10.0 µM) before and after reduction by SDT (1.0 mM). (b) Absorbance at 420 nm of IR‐C4A (10.0 µM) as a function of time following addition of SDT (1.0 mM). Inset: Absorbance spectra of IR‐C4A (10.0 µM) before and after reduction by SDT (1.0 mM). (c) Fluorescence recovery at 805 nm as a function of varying time of ICG‐C4A (1.0 µM) in the presence of rat liver microsomes (40.0 µg/mL) and NADPH (40.0 µM) under normoxic and hypoxic conditions, *λ*_ex_ = 776 nm. (d) Fluorescence recovery at 790 nm as a function of varying time of IR‐C4A (1.0 µM) in the presence of rat liver microsomes (40 µg/mL) and NADPH (40.0 µM) under normoxic and hypoxic conditions, *λ*_ex_ = 770 nm. Experimental conditions: PBS solutions, pH 7.4°C, 25 °C. All data represent the mean ± s.d. (n = 3).

### 2.4 In Vitro Cellular Imaging

The hypoxia‐responsive cellular imaging performance of the ICG‐C4A was evaluated and compared to the control groups, free ICGC and ICGC@C4A, using confocal laser scanning microscopy (CLSM) (Figure 4a,c). Under normoxic or hypoxic conditions, free ICGC exhibited no significant change in fluorescence intensity, indicating the absence of hypoxia responsiveness. In contrast, ICGC@C4A and ICG‐C4A exhibit marked increase in fluorescence intensity under hypoxic conditions compared to normoxic conditions, suggesting that ICG dye in both complexes were released from the C4A cavity because of the hypoxia‐triggered cleavage of azo units. The analytical data presented in Table 2 reveal a pronounced discrepancy in the fluorescence intensity ratio between the ICG‐C4A and the ICGC@C4A as well as the ICGC under both hypoxic and normoxic conditions. Specifically, the fluorescence intensity ratio for the ICG‐C4A was found to be 2.4‐fold higher than that of the ICGC@C4A and 8.3‐fold greater than that of the ICGC, respectively. These findings underscore the superior performance of ICG‐C4A in enhancing the signal‐to‐noise ratio, which is accompanied by an improved resistance to fluorescence leakage. Additionally, the fluorescence intensity of ICGC@C4A was higher than that of ICG‐C4A under normoxic conditions. This observation was attributed to greater leakage of ICGC from the binary inclusion complex, likely caused by biological interferents. Consequently, ICG‐C4A significantly enhances the encapsulation efficiency, leading to superior accuracy in imaging applications.

**FIGURE 4:**
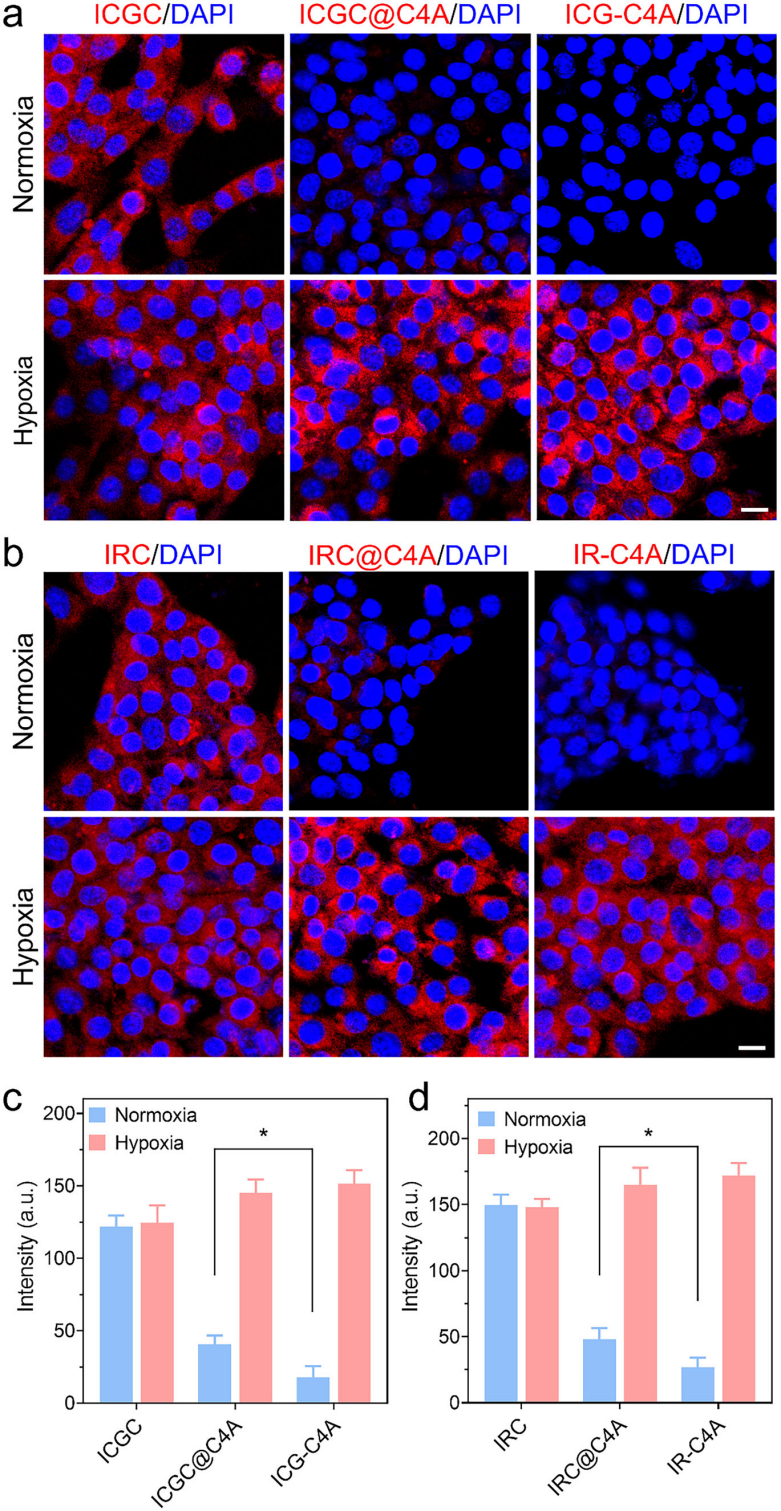
(a) Confocal laser scanning microscopy (CLSM) images of 4T1 cells incubated with ICG‐C4A and the control groups under hypoxic or normoxic conditions. (b) CLSM images of 4T1 cells incubated with IR‐C4A and the control groups under hypoxic or normoxic conditions. (c) Quantitative analysis of the fluorescence intensity of CLSM images from (a). (d) Quantitative analysis of the fluorescence intensity of CLSM images from (b). All scale bars are 50 µm. All data represent the mean ± s.d. (*n* = 3) and *p* values were calculated according to the one‐way ANOVA with Tukey's test, ^*^ *p* < 0.05.

**TABLE 2:** The fluorescence intensity ratio of two azocalixarene‐dye conjugates. and their control groups under hypoxic and normoxic conditions.

Name	Fluorescence intensity ratios(Hypoxia/Normoxia)
ICGC	1.02
ICGC@C4A	3.57
ICG‐C4A	8.45
IRC	0.99
IRC@C4A	3.43
IR‐C4A	6.39

The in vitro cellular imaging performance of IR‐C4A was also evaluated using free IRC and IRC@C4A as the control groups (Figure 4b,d). The fluorescence intensity ratios of IR‐C4A under both hypoxic and normoxic conditions were significantly higher than those of IRC@C4A and IRC, showing 1.9‐fold and 6.5‐fold enhancements, respectively. IR‐C4A consistently exhibited improved cellular imaging performance compared to both control groups.

### 2.5 In Vivo Imaging Performance

Encouraged by the results from in vitro experiments, we conducted a comprehensive evaluation of the in vivo imaging performance of ICG‐C4A and the two control groups. First, the fluorescence images as well as fluorescence intensity in the tumor sites were recorded (Figure 5a,b) at different time periods after the injection. The peak fluorescent intensities were observed at 24 h post‐injection, with the ICG‐C4A group showing nearly a 2‐fold increase in fluorescent intensity compared to that of the ICGC@C4A group, and 8‐fold increase relative to that of the ICGC group. These observations highlight a significantly enhancing imaging efficiency of the ICGC probe at the tumor sites when compared to the control groups. Subsequently, *ex* vivo imaging evaluations were further performed at 48 h after injection (Figure S30). The data analysis from Figure 5c,d demonstrates that the ICG‐C4A group exhibited the highest fluorescent intensity at the tumor sites with markedly lower fluorescence intensity in the internal organs. In contrast, the control groups showed a more diffuse fluorescence distribution, with lower contrast between tumor and internal organs. Moreover, pharmacokinetic results (Figure S29a and Table S3) showed that the elimination half‐life (*t*_eli_) of ICG‐C4A (6.1 ± 0.5 h) was significantly longer than those of ICGC (2.4 ± 0.1 h) and ICGC@C4A (4.2 ± 0.2 h), demonstrating that the ICG‐C4A can effectively prolong the blood circulation time of the ICGC, thereby providing a strong basis for its accumulation at the tumor site. These results clearly demonstrate that the self‐inclusion strategy based on azocalixarenes efficiently mitigated off‐target dissociation, improved probe stability, and imparted hypoxia‐responsiveness, leading to enhanced imaging performance of at tumor sites.

**FIGURE 5:**
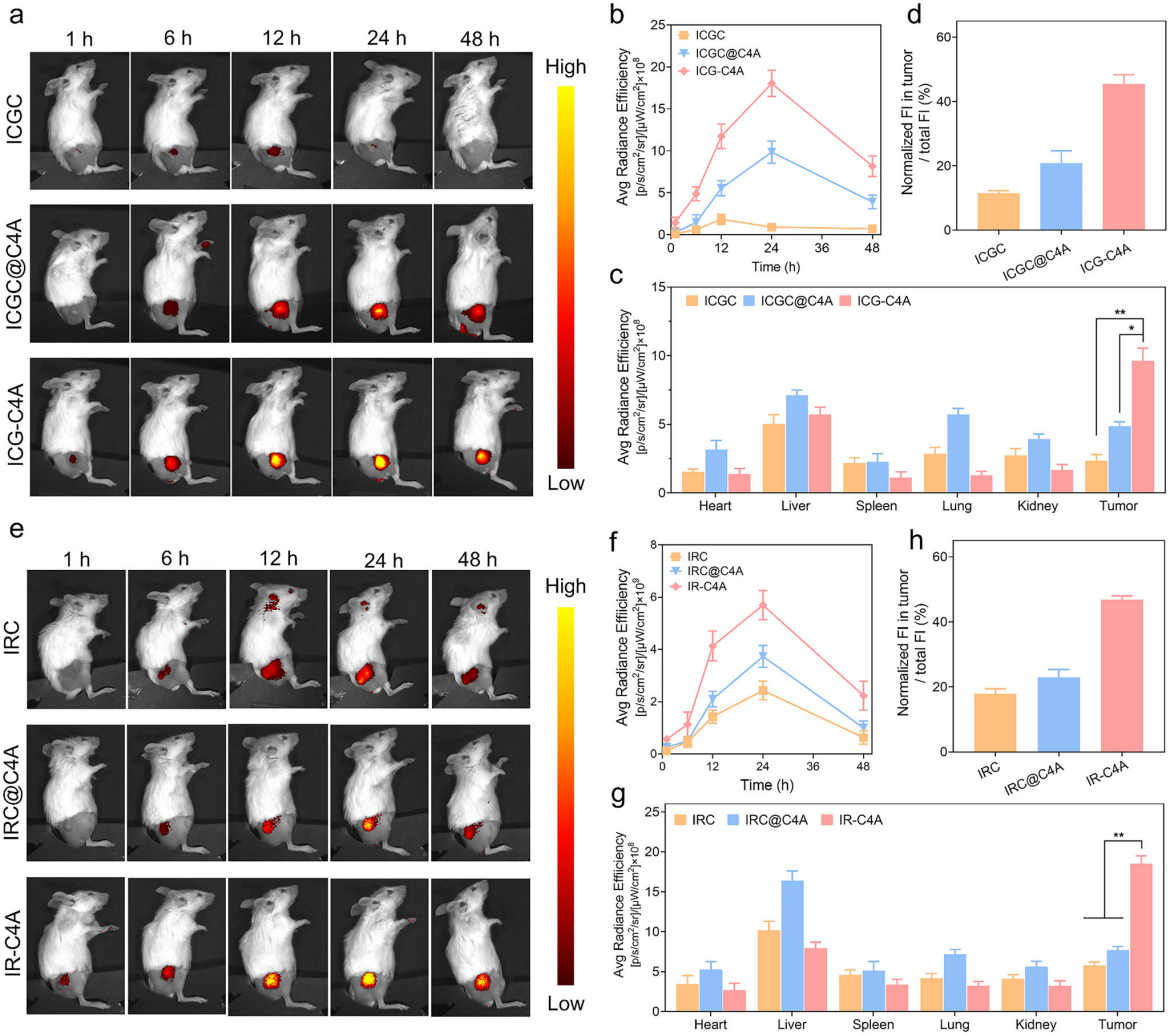
(a) In vivo fluorescence images of 4T1‐bearing mice after 1, 6, 12, 24 and 48 h intravenous injection of ICG‐C4A and the control groups, respectively. (b) Quantitative analysis of the fluorescence intensity in tumor with ICG‐C4A and the control groups at 1, 6, 12, 24, and 48 h. (c) Quantitative analysis of fluorescence intensity of ICG‐C4A and the control groups in major organs (heart, liver, spleen, lung and kidney) and tumor at 48 h. (d) Quantitative analysis of ICG‐C4A and the control group at 48 h shows the proportion of tumor fluorescence intensity in ex vivo biological samples (heart, liver, spleen, lung, kidney, and tumor). (e) In vivo fluorescence images of 4T1‐bearing mice after 1, 6, 12, 24 and 48 h intravenous injection of IR‐C4A and the control groups, respectively. (f) Quantitative analysis of the fluorescence intensity in tumor with IR‐C4A and the control groups at 1, 6, 12, 24 and 48 h. (g) Quantitative analysis of fluorescence intensity of IR‐C4A and the control groups in major organs (heart, liver, spleen, lung and kidney) and tumor at 48 h. (h) Quantitative analysis of IR‐C4A and the control group at 48 h shows the proportion of tumor fluorescence intensity in ex vivo biological samples (heart, liver, spleen, lung, kidney, and tumor). All data represent the mean ± s.d. (*n* = 3) and *p* values were calculated according to the one‐way ANOVA with Tukey's test. ^*^ *p* < 0.05, ^**^ *p* < 0.01.

Additionally, the in vivo imaging performance of the IR‐C4A were also evaluated using free IRC and IRC@C4A as the control groups, following the same experimental protocol as for ICG‐C4A. Although IRC demonstrated tumor‐targeting properties, IR‐C4A consistently exhibited increased greater tumor accumulation and higher precision compared to both control groups (Figure 5e–h). Similarly, the elimination half‐life (Figure S29b and Table S3) of IR‐C4A (7.0 ± 0.6 h) was longer than those of IRC (4.4 ± 0.3 h) and IRC@C4A (5.3 ± 0.2 h).These findings collectively demonstrate that the self‐included carrier‐dye conjugation represents a highly effective strategy for enhancing probe stability, minimizing off‐target leakage, and promoting imaging efficiency at tumor sites.

### 2.6 Biosafety Evaluation

The biosafety of ICG‐C4A was systematically evaluated using Saline, free ICGC and ICGC@C4A as the control groups. In the hematological analysis (Figure S31a,b), the blood samples of ICG‐C4A showed no significant difference compared with the saline group in the results of white blood cells (WBC), lymphocytes (Lymph), granulocyte (Gran), red blood cells (RBC), and platelets (PLT). The serum enzyme assays (Figure S31c–g) of ICG‐C4A showed no significant difference in the results of alanine aminotransferase (ALT), aspartate aminotransferase (AST), alkaline phosphatase (ALP), blood urea nitrogen (BUN), and creatinine (CREA) compared with the Saline group. These observations indicated that ICG‐C4A groups possess good biosafety, and no obvious hepatorenal toxicity. Furthermore, histopathological analysis of the main internal mice organs showed no clear organ lesions in the ICG‐C4A (Figure S32) which verifies that they have excellent biosafety.

The biosafety of IR‐C4A was also evaluated using Saline, free IRC and IRC@C4A as the control groups. IR‐C4A demonstrated no significant differences compared to the Saline group in hematological, serum enzyme assays, and histopathological of visceral tissues (Figures S33 and S34). These consistent findings across multiple safety evaluation endpoints indicate that IR‐C4A exhibits excellent biocompatibility. Collectively, these results demonstrate that the self‐inclusion fluorescent probes based on azocalixarenes possesses a favorable biosafety profile.

## 3 Conclusion

Self‐Included Fluorescent Probes (SIFPs), based on self‐included macrocyclic carrier‐dye conjugates, present a highly promising strategy for developing activatable fluorescent probes. By employing azocalixarenes as the macrocyclic carrier, we successfully achieved effective encapsulation and protection of covalently linked fluorescent probes. Comprehensive in vitro and in vivo experiments comparing the SIFPs with free fluorescent probes and binary carrier‐dye complexes demonstrated their ability to prevent premature leakage and enhance probe stability. Moreover, the hypoxia‐responsive nature of azocalixarenes enabled precise activation of the fluorescent probes, significantly improving in vivo imaging performance at tumor sites. Thus, SIFPs combine the advantages of both covalent and non‐covalent strategies. Given the abundance of existing fluorescent probes and the wide availability of diverse supramolecular hosts, the SIFPs strategy offers a convenient, effective, and versatile approach for converting current fluorescent dyes into activatable probes with superior stability. This proof‐of‐concept study not only underscores the potential of SIFPs for imaging applications but also holds promise for advancing the development of clinically relevant imaging probes.

## Author Contributions

**Shun‐Yu Yao**: Conceptualization, Funding acquisition, Writing – original draft, Writing – review & editing; **Ze‐Han Wang**: Methodology, Conceptualization, Validation, Writing – review & editing; **Chenyin He**: Validation, Investigation; **Lidian Zheng**: Validation, Investigation; **An‐Kang Ying**: Conceptualization, Investigation; **Kang Cai**: Conceptualization, Supervision; **Ai‐Feng Liu**: Conceptualization, Supervision; **Xiufeng Xiao**: Conceptualization, Funding acquisition, Writing – review & editing, Supervision; **Dong‐Sheng Guo**: Writing – review & editing, Supervision.

## Conflicts of Interest

The authors declare no conflicts of interest.

## Supporting information




**Supporting File**: advs78729‐sup‐0001‐SuppMat.docx.

## Data Availability

The data that supports the findings of this study are available in the supplementary material of this article.
