# Supplementary material for: Self‐Included Fluorescent Probes via Azocalix[4]arene–Dye Conjugation for Activatable and Stable NIR Imaging
Source: Adv Sci (Weinh). 2026 Nov 9:e78729. Online ahead of print. doi: 10.1002/advs.78729 (PMC13749010; doi:10.1002/advs.78729)
Supplement: Supplementary file 1 [file ADVS-9999-e78729-s001.docx]

**Supporting Information**

**Self-Included Fluorescent Probes via Azocalix[4]arene–Dye Conjugation for Activatable and Stable NIR Imaging**

Shun-Yu Yao^[a]^*^+^, Ze-Han Wang^[c]+^, Chenyin He^[a]^, Lidian Zheng^[a]^, An-Kang Ying^[c]^, Kang Cai^[c]^, Ai-Feng Liu^[b]^*, Xiufeng Xiao^[a]^*, Dong-Sheng Guo^[c]^

[a]	College of Chemistry and Materials Science, Fujian Provincial Key Laboratory of Advanced Materials Oriented Chemical Engineering, Fujian Normal University, Fuzhou, 350007, China.

[b]	Orthopedics Department, First Teaching Hospital of Tianjin University of Traditional Chinese Medicine, National Clinical Research Center for Chinese Medicine Acupuncture and Moxibustion, Tianjin, 300381, China

[c]	College of Chemistry, State Key Laboratory of Medicinal Chemical Biology, Key Laboratory of Functional Polymer Materials (Ministry of Education), Frontiers Science Center for New Organic Matter, Collaborative Innovation Center of Chemical Science and Engineering (Tianjin), Nankai University, 300071 Tianjin, China.

[*]	Corresponding Author(s): Shun-Yu Yao, syyao@fjnu.edu.cn; Ai-Feng Liu, draifeng@163.com; Xiufeng Xiao, xfxiao@fjnu.edu.cn.

[+]	These authors contributed equally: Shun-Yu Yao, Ze-Han Wang

Keywords: Fluorescent probes; Azocalixarene; Precise activation; Hypoxia-responsive; Self-inclusion

contents

[1. Materials and methods	3](#_Toc244175076)

[1.1 Materials	3](#_Toc244175077)

[1.2 Apparatus	3](#_Toc244175078)

[1.3 Binding affinities of CSAC4A towards guests	3](#_Toc244175079)

[1.4 Binding constants (*K*_self_) of the self-inclusion complexes	4](#_Toc244175080)

[1.5 Data analysis of fluorescence titrations	4](#_Toc244175081)

[1.6 Travelling Wave Ion Mobility Spectrometry–Mass Spectrometry (TWIMS–MS)	4](#_Toc244175082)

[1.7 Evaluation of the stability	4](#_Toc244175083)

[1.8 Evaluation of the hypoxia-responsive behaviors	5](#_Toc244175084)

[1.9 Cell culture and *In vitro* cellular fluorescence imaging	5](#_Toc244175085)

[1.10 Mice	5](#_Toc244175086)

[1.11 *In vivo* fluorescence imaging	6](#_Toc244175087)

[1.12 Pharmacokinetics of different groups	6](#_Toc244175088)

[1.13 Statistical analysis	6](#_Toc244175089)

[2. Synthetic procedure of the self-included complexes and the control compounds	7](#_Toc244175090)

[2.1 Synthesis of ICG-C4A	7](#_Toc244175091)

[2.2 Synthesis of IR-C4A	8](#_Toc244175092)

[2.3 Synthesis of ICGA	9](#_Toc244175093)

[2.4 Synthesis of IRA	10](#_Toc244175094)

[3. Supporting results and experimental raw data	11](#_Toc244175095)

[3.1 Structural and molecular weight characterization of compounds	11](#_Toc244175096)

[3.2 Collision cross section (CCS)	20](#_Toc244175097)

[3.3 Binding affinities of self-inclusion	21](#_Toc244175098)

[3.4 UV-Vis absorption of dyes	22](#_Toc244175099)

[3.5 Hypoxia-responsive behaviors	25](#_Toc244175100)

[3.6 Pharmacokinetics	27](#_Toc244175101)

[3.7 *Ex vivo* fluorescence imaging	28](#_Toc244175102)

[3.8 *In vivo* safety evaluation	28](#_Toc244175103)

[References	30](#_Toc244175104)

# 1. Materials and methods

**1.1 Materials**

The reagents and solvents mentioned in this article were purchased from commercial sources. Unless otherwise specified, the purchased reagents and solvents were used directly without further purification. 4-Aminobenzoic acid, 4-aminobenzenesulfonic acid, sodium acetate trihydrate, 2,3,3-trimethylindolenine, Indocyanine green (ICG), IR780 ethyl iodide (IR780), bromoacetic acid, bromoethane, sodium nitrite (NaNO_2_), and sodium carbonate (Na_2_CO_3_) were purchased from Shanghai Aladdin. N-Boc-2-(2-aminoethoxy)ethanamine were purchased from Shanghai Bide. Triethylamine (Et_3_N), trifluoroacetic acid (TFA), 1-(3-Dimethylaminopropyl)-3-ethylcarbodiimide (EDC), N-Hydroxysuccinimide (NHS), 2-(7-azabenzotriazole)-N,N,N',N'-tetramethylurea hexafluorophosphate (HATU), and N,N-Diisopropylethylamine (DIPEA) were purchased from McLean. Glutacondianil hydrochloride and 2-chloro-3-(hydromethylene)cyclohex-1-enecarbaldehyde were purchased from Shanghai Merrill. Acetic acid (AcOH), pyridine, sodium acetate (NaOAc), 1-butanol, N,N-Dimethylformamide (DMF), methanol (MeOH), and ethyl acetate (EA) were purchased from Beijing J&K. Ethanol (EtOH), acetic anhydride (Ac_2_O), Sodium dithionite (SDT), and toluene were purchased from Tianjin Hedong Guangda Service Department. Penicillin/streptomycin, fetal bovine serum (FBS), and DMEM medium (DMEM) were purchased from Gibco.

## 1.2 Apparatus

^1^H NMR and ^13^C NMR spectra were measured using Bruker nuclear magnetic resonance spectroscopy instrument. Mass spectra were recorded using an Agilent 6540 TOF (ESI). All trapped ion mobility spectrometry (TIMS) measurements were performed with a Waters Vion IM-QTOF instrument. Steady-state fluorescence measurements were recorded in a conventional quartz cell (light path 10 mm) on a Cary Eclipse spectrophotometer equipped with a Cary single-cell peltier accessory. UV–Vis spectra were recorded in a quartz cell (light path 10 mm) on a Cary 100 UV-Vis spectrophotometer equipped with a Cary dual-cell peltier accessory. A microplate reader (Thermo Scientific Multiskan FC) was used for cell viability analysis. Confocal laser scanning microscopy (CLSM) imaging was performed using a Nikon A1R+ system. *In vivo* distribution of fluorescence was imaged by the IVIS Lumina imaging system (Caliper Life Sciences, USA).

## 1.3 Binding affinities of CSAC4A towards guests

The binding constants (*K*_a_) of C4A towards ICGC and IRC were determined by competitive fluorescent titrations using rhodamine B (RhB) as the reporter dye. Firstly, the *K*_a_ values between RhB and C4A were measured using direct fluorescent titrations: the fluorescence intensity of 0.50 μM RhB in phosphate buffered saline (PBS) solution (pH = 7.4) was recorded, then C4A was gradually added into the solution to quench the fluorescent of RhB, and the *K*_a_ between C4A and RhB was determined according to the 1:1 binding model. The competitive fluorescent titrations were taken by gradually adding the competitor (ICGC/IRC) to the solutions containing the reporter pairs (RhB and C4A) whose association constants were already known. The association constants between competitors and C4A were obtained by fitting fluorescent intensities at their emission wavelengths and concentrations of the drugs according to the 1:1 competitive binding model.

## 1.4 Binding constants (*K*_self_) of the self-inclusion complexes

The *K*_self_ of self-inclusion complexes were determined by fluorescent titrations using silicon (IV) phthalocyanine (SiPcN_2_) as the reporter dye. Firstly, the *K*_a_ (SiPcN_2_@C4A) value between SiPcN_2_ and C4A was measured by direct fluorescent titrations: the fluorescence intensity of 0.50 μM SiPcN_2_ in PBS solution (pH = 7.4) was recorded, then C4A was gradually added into the solution to quench the fluorescent of SiPcN_2_, and the *K*_a_ (SiPcN_2_@C4A) was determined according to the 1:1 binding model. Subsequently, the *K*_rel_ of self-inclusion complexes towards SiPcN_2_ was determined according to the above direct titration method, with the SiPcN_2_ concentration of 0.50 μM. Since the single functional group modification of azocalixarene will not significantly alter its cavity properties, the calculation formula of self-inclusion binding constants (*K*_self_) is as follows:

## 1.5 Data analysis of fluorescence titrations

The fitting of data from direct host-guest titrations and competitive titrations was performed in a nonlinear manner^[1]^ and the fitting modules were downloaded from the website of Prof. Nau’s group (http://www.jacobs-university.de/ses/wnau) under the column of “Fitting Functions”.

## 1.6 Travelling Wave Ion Mobility Spectrometry–Mass Spectrometry (TWIMS–MS)

Ion mobility spectrometry (IMS) is a technique that can be coupled to mass spectrometry (MS). IMS provides collision cross sections (CCSs) of ions and thus insights into their gas-phase structures. All IMS measurements were performed with a Waters Vion IMQTOF instrument. Source conditions for electrospraying the ions in IMS were set as follows: capillary voltage, 1.5 kV; cone voltage, 60 V; source offset voltage, 80 V; source temperature, 120 °C; desolvation temperature, 500 °C; cone gas flow rate (N_2_), 50 L/h; and desolvation gas flow rate (N_2_), 800 L/h. Calibration of CCS was conducted according to the manufacture′s guidelines using a mixture of calibrants. Data acquisition was controlled by the UNIFITM 1.9.3.0 software (Waters). The CCS values were determined using a standard calibrant-based calibration approach: 1) Acquisition of raw data. All ion mobility spectrometry (IMS) measurements were performed on a Waters Vion IM-QTOF instrument using nitrogen as the buffer gas. Each ion yields a specific drift time ($t_{d}$, ms) in the mobility cell. Simultaneously, high-resolution mass spectrometry provides the accurate mass-to-charge ratio (m/z) and charge state (z). 2) Construction of the calibration curve. Poly-DL-alanine (a calibrant purchased from Waters with known nitrogen CCS values, Ω) was used as the calibrant. For each calibrant ion, its drift time ($t_{d}$) was measured, and a correction factor was calculated as $\Omega^{'}=\Omega\times\sqrt{\mu}/z$, where$\mu$is the reduced mass of the ion–nitrogen pair. Using Waters UNIFITM software, $\Omega^{'}$ was fitted to $t_{d}$by a power-law function, giving the calibration equation $\Omega^{'}=A\times(t_{d})^{B}$. Under our experimental conditions, the goodness-of-fit $R^{2}$ was greater than 0.99. 3) Calculation of CCS for analytes. For each analyte, its m/z and z were confirmed from the mass spectrum, and $\mu$ was calculated. The measured drift time ($t_{d}$) was substituted into the above calibration equation to obtain $\Omega^{'}$, and the final collision cross section was derived as $\Omega=(\Omega^{'}\times z)/\sqrt{\mu}$.

## 1.7 Evaluation of the stability

The chemical stability of ICGC, ICGA, ICGC@C4A and ICG-C4A in PBS solution was examined both in the presence and absence of 10% FBS. Samples (10.0 μM) were stored in the dark at 25 °C for 96 h. At predetermined times, UV–vis absorption spectra of the solutions were measured to evaluate the chemical stability of ICG-C4A and the control groups. Each sample was measured for three times. Evaluate the chemical stability of IRC, IRA, IRC@C4A and IR-C4A as described above.

To evaluate the photostability, ICGC, ICGA, ICGC@C4A and ICG-C4A (10.0 μM, in PBS, pH = 7.4) was exposed to 808 nm wavelength laser (1 W cm^-2^) for 8 min. UV–vis absorption tests were measured every 60 s, and the time-dependent decay of the ICGC normalized absorbance was used to indicate changes in the maximum absorption value. Each sample was measured three times. The photostability of IRC, IRA, IR@C4A, and IR-C4A was evaluated according to the aforementioned method.

## 1.8 Evaluation of the hypoxia-responsive behaviors

For ICG-C4A (10.0 μM) or IR-C4A (10.0 μM), upon addition of SDT (1.0 mM), the absorbance at 420 nm as a function of time was measured using a UV/Vis spectrophotometer. To investigate hypoxia-triggered dye release, rat liver microsomes (40 μg/mL) and NADPH (40.0 μM) were added to ICG-C4A (1.0 μM) or IR-C4A (1.0 μM), and the corresponding fluorescence spectra were recorded under hypoxic and normoxia conditions. For hypoxic measurements, PBS solutions were pre-bubbled with a gas mixture (1% O_2_, 5% CO_2_, 94% N_2_) for 2 hours to deplete dissolved oxygen, followed by the sequential addition of rat liver microsomes, NADPH, and the ICG-C4A or IR-C4A probes. Throughout the measurement, the cuvette was maintained under a positive pressure of the same hypoxic gas mixture using a balloon, with continuous stirring to ensure gas-liquid equilibrium. Normoxic controls were performed under ambient atmospheric conditions.

ICG-C4A was incubated with 4T1 cells under hypoxic conditions for 4 h. The cells were then lysed and centrifuged to collect the supernatant. Methanol was added to the supernatant to precipitate water-soluble proteins and other impurities or salts. After a second centrifugation, the supernatant was collected and filtered through a 0.22 μm organic-phase filter membrane for subsequent UPLC-MS/MS analysis (Waters APGC/UPLC Xevo TQ-S, ACQUITY UPLC BEH C18 column, 1.7 μm, 2.1 mm × 100 mm, 50 °C). For NH_2_C4A: m/z 485.22 → m/z 468.19, m/z 485.22 → m/z 451.17. For ICGC-NH_2_: m/z 772.42 → m/z 645.33, m/z 772.42 → m/z 574.28.

## 1.9 Cell culture and *In vitro* cellular fluorescence imaging

The 4T1 cells were purchased from the Cell Bank of the Chinese Academy of Sciences (Shanghai, China). 4T1 cells were cultured in DMEM medium containing FBS (10%) and penicillin/streptomycin (1%). When the cell density reached 70 − 80%, trypsin solutions was added and incubated for 5 min to separate the cells. After centrifugation at 1000 rpm for 5 min, the supernatant was discarded. Add a portion of serum-supplemented medium to neutralize residual trypsin. Cells were suspended in serum-supplemented medium and incubated at 37 °C and 5% CO_2_.

4T1 cells (1 × 10^5^ cells per well) were seeded and cultured in confocal microscopic dishes. After adherence, the culture medium was replaced with fresh medium containing ICGC, ICGC@C4A and ICG-C4A (10.0 μM for ICGC). The cells were incubated in normoxia for 6 h. After incubation, discarded and replaced the medium. Part of the confocal microscopic dishes was transferred to a hypoxic chamber and incubated for 12 h under normoxia or hypoxia. Cells were washed three times with PBS before imaging by confocal laser scanning microscope. The *In vitro* cellular fluorescence imaging of IRC, IRC@C4A, and IR-C4A were evaluated according to the aforementioned method.

## 1.10 Mice

Female BALB/c mice (6-8 weeks old) were purchased from Shanghai Slaccas Animal Technology Company. All animal experiments conducted in this study were approved by the Animal Ethical and Welfare Committee of Fujian Normal University (Approval Number: IACUC-20250011) and were performed in accordance with the guidelines outlined in the National Institutes of Health guide for the care and use of laboratory animals.

## 1.11 *In vivo* fluorescence imaging

To establish the 4T1 tumor-bearing mouse model, 1 × 10^6^ 4T1 cells were injected subcutaneously into the right hind limb of BALB/c mice. The mice with tumor volumes around 200 mm³ were randomized into 3 groups and injected intravenously with ICGC, ICGC@C4A, or ICG-C4A at 5.0 mL/kg. ICGC and ICGC@C4A were administered at an ICGC-equivalent concentration of 200.0 μM, while ICG-C4A was administered at 200.0 μM based on its own molecular weight. *In vivo* fluorescence imaging of ICG-C4A and its control groups were imaged by IVIS Lumina imaging system at the time of 1h, 6 h, 12 h, 24 h and 48 h after injection, respectively. Fluorescent images were analyzed using Living Image 3.1. For the *ex vivo* tissues distribution study, the mice were sacrificed after 24 h injection, and tumors as well as major organs were collected and subjected for *ex vivo* imaging. The *In vivo* fluorescence imaging performance of IRC, IRC@C4A, and IR-C4A were evaluated according to the aforementioned method.

## 1.12 Pharmacokinetics of different groups

Healthy SD rats were randomly divided into 8 groups (n = 3 per group) corresponding to different blood sampling time points. Free ICGC, ICGC@C4A, and ICG-C4A were administered via a single intravenous injection at a dosing volume of 1.0 mL. The doses of ICGC and ICGC@C4A were set at an equivalent ICGC dose of 1.0 mg/kg, while ICG-C4A was administered at a dose of 3.1 mg/kg (equimolar to the ICGC dose). At the indicated time points 5 min, 15 min, 30 min, 1 h, 2 h, 4 h, 12 h and 24h, blood samples (500 μL) were collected from the orbit using a 0.5-mm capillary tube. The water-soluble protein as well as other impurities or salts in plasma were precipitated out by methanol. The supernatants were collected and passed through a 220 μm organic phase filter membrane, followed by adding 1.00 μM ICGC or ICG-C4A (internal standard) for further LC-MS analysis (Agilent 1290II-6460). The chromatographic separation was accomplished with the mobile phase consisting of acetonitrile and water (containing 0.2% formic acid, *V*/*V*) in gradient elution ranging from 5 : 95 to 100 : 0 (acetonitrile/water, *V*/*V*). The flow rate of the mobile phase was set at 0.30 mL min^−1^ and the injection volume was 5.00 μL. Concentration of ICGC or ICG-C4A were determined according to the standard curve, respectively. For the IR-C4A series, the same experimental protocol was followed, except that IRC and IRC@C4A were dosed at an equivalent IRC dose of 1.0 mg/kg, and IR-C4A was administered at 3.2 mg/kg (equimolar to the IRC dose).

## 1.13 Statistical analysis

Data are presented as the mean ± standard deviation. Statistical analysis of data was performed with one-way analysis of variance (ANOVA) for comparison of multiple groups using the GraphPad Prism 8.0 software. The level of significance was defined at **p* < 0.05, ***p* < 0.01 and ****p* < 0.001.

# 2. Synthetic procedure of the self-included complexes and the control compounds

## 2.1 Synthesis of ICG-C4A

**Scheme S1**. The synthetic routes of ICG-C4A.

**2.1.1 Synthesis of C4A-NH_2_**

CSAC4A was synthesized according to the protocols reported in literature.^[2]^ CSAC4A (200 mg, 0.18 mmol) was dissolved in DMF (20 mL), DIPEA (44 mg, 0.34 mmol) and HATU (78 mg, 0.21 mmol) were sequentially added to the DMF solution, and stirred at room temperature for 30 min. N-Boc-2-(2-Aminoethoxy) ethanamine (56 mg, 0.27 mmol) was added to the reaction system, and the mixture was stirred at room temperature for 12 h. Removing the solvents *in vacuo* to obtain the crude product of CSAC4A-BOC. The above-mentioned crude product was re-dissolved in DMSO (20 mL), and then TFA (1.5 mL) was added to the solution, followed by stirring at room temperature for 6 h. After removing the solvents *in vacuo*, the crude product of CSAC4A-NH_2_ was recrystallized in water and methanol to obtain 205 mg of brick-red powder with a yield of 89%.^1^H NMR (400 MHz, DMSO-*d*_6_) δ: 7.94 (d, *J* = 8.5 Hz, 2H), 7.85 (t, *J* = 6.9 Hz, 10H), 7.74 (d, *J* = 5.7 Hz, 12H), 4.11 (m, 8H), 3.59 – 3.37 (m, 8H), 2.86 (s, 2H) ppm. Mass spectrum (ESI): [M+H]^+^: *m/z* calcd. for (C_57_H_51_N_10_O_15_S_3_^+^): 1211.26920, found: 1211.26817.

**2.1.2 Synthesis of ICG-COOH**

ICG-COOH was synthesized according to the protocols reported in literature.^[3]^ In briefly, 1,1,2-trimethyl-3-(carboxymethyl)-1H-benzo[e]indole-3-ium bromide (352 mg, 1.01 mmol), Glutacondianil hydrochloride (288 mg, 1.01 mmol), acetic acid (5 mL) and acetic anhydride (5 mL) were added sequentially to a 25 mL round-bottomed flask. After mixing evenly, the reaction was refluxed for 4 hours. The solvent was spin-dried *in vacuo* to obtain the crude products. In another 25 mL round-bottomed flask, the crude products (390 mg), 3-ethyl-1, 1, 2-trimethyl-1H-benzo[e]indol-3-ium iodide (360 mg, 1.01 mmol), pyridine (5 mL) and acetic acid (5 mL) were mixed together and refluxed for 2 hours. The solvent was removed *in vacuo*, and the ICG-COOH was separated by column chromatography (*V*:*V*, dichloromethane: methanol = 30:1) to obtain 366 mg with a yield of 36%.^1^H NMR (400 MHz, DMSO-*d*_6_) δ: 8.26 (d, *J* = 4.4 Hz, 2H), 8.07 (dd, *J* = 7.2, 4.2 Hz, 4H), 7.99 (q, *J* = 7.3, 6.7 Hz, 2H), 7.81 (t, *J* = 6.3 Hz, 1H), 7.73 (t, *J* = 4.9 Hz, 2H), 7.65 (t, *J* = 4.0 Hz, 2H), 7.50 (t, *J* = 3.9 Hz, 2H), 6.59 (q, *J* = 6.4 Hz, 2H), 6.42 (q, *J* = 6.2 Hz, 2H), 4.25 (q, *J* = 3.5 Hz, 2H), 3.73 (s, 2H), 1.91(s, 12H), 1.34 (t, *J* = 3.6 Hz, 3H) ppm. Mass spectrum (ESI): [M-Br+H_2_O]^+^: *m/z* calcd. for (C_39_H_40_N_2_O_3_^+^): 585.31117, found: 585.31243.

**2.1.3 Synthesis of ICG-C4A**

ICG-COOH (145 mg, 0.25 mmol) was dissolved in DMF (20 mL), EDC (78 mg, 0.50 mmol) and NHS (58 mg, 0.50 mmol) were sequentially added to the DMF solution, and stirred at room temperature for 30 min. CSAC4A-NH_2_ (260 mg, 0.23 mmol) was added to the reaction system, and the mixture was stirred at room temperature for 12 h. After removing the solvents *in vacuo*, the crude product was recrystallized in ethyl acetate and methanol to obtain 334 mg with a yield of 80%.^1^H NMR (400 MHz, DMSO-*d*_6_) δ: 8.57 (s, 1H), 8.22 (t, *J* = 3.6 Hz, 2H), 8.06 – 7.89 (m, 8H), 7.79 (d, *J* = 5.0 Hz, 11H), 7.69 (d, *J* = 2.6 Hz, 14H), 7.63 – 7.60 (m, 2H), 7.47 (d, *J* = 4.3 Hz, 2H), 6.81 (s, 1H), 6.64 (t, *J* = 6.3 Hz, 2H), 6.44 (dt, *J* = 12.6, 6.5 Hz, 2H), 4.23 (s, 2H), 3.69 (s, 8H), 3.50 (t, *J* = 3.0 Hz, 2H), 3.40 (t, *J* = 3.0 Hz, 4H), 3.24 (s, 2H), 3.07 (s, 2H), 1.88 (d, *J* = 5.2 Hz, 12H), 1.30 (d, *J* = 3.6 Hz, 3H). ppm. ^13^C NMR (100 MHz, DMSO-*d*_6_) δ: 174.81, 173.80, 169.60, 168.50, 160.40, 154.95, 152.76, 150.59, 148.86, 145.39, 143.69, 138.00, 136.69, 135.40, 133.81, 132.89, 130.40, 128.69, 127.60, 126.29, 124.90, 123.40, 122.89, 121.50, 120.26, 119.77, 118.26, 114.30, 105.20, 104.09, 70.20, 62.50, 54.40, 49.60, 46.40, 40.13, 39.92, 39.71, 39.50, 39.29, 39.08, 38.88, 29.89, 27.39, 26.00, 15.09 ppm. Mass spectrum (ESI): [M+2NH_3_−2H]^2−^: *m/z* calcd. for (C_96_H_90_N_14_O_16_S_3_^2−^): 895.79329, found: 895.79415.

## 2.2 Synthesis of IR-C4A

**Scheme S2**. The synthetic routes of IR-C4A.

**2.2.1 Synthesis of IR-COOH**

According to the protocols reported in literature.^[4]^ In briefly, 1-(carboxymethyl)-2,3,3-trimethyl-3H-indol-1-ium (563 mg, 1.89 mmol), 2-chloro-1-formyl-3-hydroxymethylenecyclohexene (380 mg, 2.20 mmol), Sodium acetate (180 mg, 2.20 mmol) and ethanol (25 mL) were added sequentially to a 50 mL round-bottomed flask. After mixing evenly, the reaction was refluxed for 2 hours at 70 °C. The solvent was removed *in vacuo*, and the crude products (red powder) were separated by column chromatography (*V*:*V*, Dichloromethane: Methanol = 30:1). In another 25 mL round-bottomed flask, the above-mentioned red powder crude products (316 mg), 1-ethyl-2, 3, 3-trimethylindolenium Iodide (596 mg, 1.89 mmol), toluene (7 mL) and 1-butanol (5 mL) were mixed together and refluxed for 8 hours at 120 °C. The solvent was removed *in vacuo*, and the IR-COOH was separated by column chromatography (*V*:*V*, dichloromethane: methanol = 30:1) to obtain 460 mg with a yield of 46%.^1^H NMR (400 MHz, DMSO-*d*_6_) δ: 8.30 – 8.22 (m, 2H), 7.64 (t, *J* = 3.2 Hz, 2H), 7.47 – 7.42 (m, 4H), 7.33 – 7.26 (m, 2H), 6.32 (t, *J* = 7.3 Hz, 2H), 4.25 (q, *J* = 3.7 Hz, 2H), 3.69 (s, 2H), 2.72 (q, *J* = 2.6 Hz, 4H), 1.86 (p, *J* = 3.2 Hz, 2H), 1.67 (q, *J* = 0.9 Hz, 12H), 1.31 (t, *J* = 3.6 Hz, 3H) ppm. Mass spectrum (ESI): [M-Cl]^+^: *m/z* calcd. for (C_34_H_38_ClN_2_O_2_^+^): 541.26163, found: 541.26218.

**2.2.2 Synthesis of IR-C4A**

The synthesis method of CSAC4A-NH_2_ is detailed in section 2.1.1, and will not be further elaborated here.

IR-COOH (136 mg, 0.25 mmol) was dissolved in DMF (20 mL), EDC (78 mg, 0.50 mmol) and NHS (58 mg, 0.50 mmol) were sequentially added to the DMF solution, and stirred at room temperature for 30 min. CSAC4A-NH_2_ (260 mg, 0.23 mmol) was added to the reaction system, and the mixture was stirred at room temperature for 12 h. After removing the solvents *in vacuo*, the crude product was recrystallized in ethyl acetate and methanol to obtain 310 mg with a yield of 75%.^1^H NMR (400 MHz, DMSO-*d*_6_) δ: 8.57 (t, *J* = 2.8 Hz, 1H), 8.23 (dt, *J* = 7.5, 4.0 Hz, 2H), 7.93 (d, *J* = 4.3 Hz, 2H), 7.84 – 7.76 (m, 10H), 7.74 – 7.66 (m, 12H), 7.59 (t, *J* = 3.5 Hz, 2H), 7.41 (dd, *J* = 3.8, 2.6 Hz, 4H), 7.28 – 7.23 (m, 2H), 6.83 (s, 1H), 6.40 – 6.33 (m, 2H), 4.21 (q, *J* = 4.3 Hz, 2H), 3.68 (s, 8H), 3.50 (t, *J* = 3.0 Hz, 2H), 3.40 (t, *J* = 3.0 Hz, 4H), 3.24 (s, 2H), 3.06 (d, *J* = 2.8 Hz, 2H), 2.72 – 2.65 (m, 4H), 1.86 – 1.80 (m, 2H), 1.65 (d, *J* = 3.3 Hz, 12H), 1.28 (t, *J* = 3.6 Hz, 3H) ppm. ^13^C NMR (100 MHz, DMSO-*d*_6_) δ: 173.81, 171.37, 166.17, 165.32, 162.80, 158.85, 154.23, 152.52, 149.63, 145.09, 143.28, 141.43, 140.38, 139.55, 138.52, 135.71, 134.81, 131.12, 130.21, 128.92, 128.12, 127.55, 126.95, 125.94, 125.30, 124.20, 123.51, 122.83, 121.94, 114.47, 111.86, 108.82, 103.81, 102.32, 69.20, 56.90, 54.94, 50.40, 49.30, 36.67, 36.27, 34.58, 28.68, 27.76, 25.73, 25.01, 18.84, 16.11, 14.56 ppm. Mass spectrum (ESI): [M+CH_3_COOH−3H]^2−^: *m/z* calcd. for (C_93_H_87_ClN_12_O_18_S_3_^2−^): 895.75782, found: 895.75776.

## 2.3 Synthesis of ICGA

**Scheme S3**. The synthetic routes of ICGA.

Azophenol-COOH (63 mg, 0.25 mmol) was dissolved in DMF (15 mL), DIPEA (65 mg, 0.50 mmol) and HATU (104 mg, 0.28 mmol) were sequentially added to the DMF solution, and stirred at room temperature for 30 min. N-Boc-2-(2-Aminoethoxy) ethanamine (62 mg, 0.30 mmol) was added to the reaction system, and the mixture was stirred at room temperature for 12 h. The above-mentioned crude product was re-dissolved in DMSO (20 mL), and then TFA (1.5 mL) was added to the solution, followed by stirring at room temperature for 6 h. After removing the solvents in vacuo, the crude product of Azophenol-PEG_1_-NH_2_ was recrystallized in water and methanol to obtain 56 mg of yellow-brown powder with a yield of 69%.^1^H NMR (600 MHz, DMSO-*d*_6_) δ: 10.54 (s, 1H), 8.65 (t, *J* = 2.8 Hz, 1H), 8.03 (d, *J* = 4.1 Hz, 2H), 7.91(s, 2H), 7.88 (d, *J* = 4.1 Hz, 2H), 7.84 (d, *J* = 4.2 Hz, 2H), 6.98 (d, *J* = 4.2 Hz, 2H), 3.63 (dt, *J* = 9.0, 2.7 Hz, 4H), 3.51 (q, *J* = 2.9 Hz, 2H), 3.02 (t, *J* = 2.6 Hz, 2H) ppm.

ICG-COOH (145 mg, 0.25 mmol) was dissolved in DMF (20 mL), EDC (78 mg, 0.50 mmol) and NHS (58 mg, 0.50 mmol) were sequentially added to the DMF solution, and stirred at room temperature for 30 min. Azophenol-PEG_1_-NH_2_ (78 mg, 0.23 mmol) was added to the reaction system, and the mixture was stirred at room temperature for 12 h. After removing the solvents *in vacuo*, the crude product was recrystallized in water and methanol to obtain 172 mg with a yield of 85%.^1^H NMR (400 MHz, DMSO-*d*_6_) δ: 10.40 (s, 1H), 8.63 (t, *J* = 2.8 Hz, 1H), 8.26 (d, *J* = 4.2 Hz, 2H), 8.08 – 7.95 (m, 8H), 7.88 – 7.76 (m, 5H), 7.74 – 7.70 (m, 2H), 7.65 (t, *J* = 4.0 Hz, 2H), 7.52 – 7.48 (m, 2H), 6.96 (d, *J* = 4.4 Hz, 2H), 6.77(s, 1H), 6.67 – 6.59 (m, 2H), 6.51 – 6.42 (m, 2H), 4.24 (q, *J* = 3.4 Hz, 2H), 3.54 (t, *J* = 2.9 Hz, 2H), 3.46 – 3.40 (m, 4H), 3.28 (s, 2H), 3.09 (q, *J* = 3.0 Hz, 2H), 1.91 (s, 12H), 1.33 (t, *J* = 3.5 Hz, 3H) ppm. ^13^C NMR (100 MHz, DMSO-*d*_6_) δ: 175.92, 173.12, 172.48, 169.02, 160.40, 155.71, 146.82, 143.32, 141.46, 136.62, 132.01, 126.53, 124.02, 119.91, 115.42, 111.88, 102.33, 100.72, 71.91, 64.82, 57.92, 40.62, 40.20, 39.78, 33.57, 28.62, 27.78, 21.54 ppm. Mass spectrum (ESI): [M]^+^: *m/z* calcd. for (C_56_H_57_N_6_O_4_^+^): 877.44358, found: 877.44276.

## 2.4 Synthesis of IRA

**Scheme S4**. The synthetic routes of IRA.

Azophenol-COOH (63 mg, 0.25 mmol) was dissolved in DMF (15 mL), DIPEA (65 mg, 0.50 mmol) and HATU (104 mg, 0.28 mmol) were sequentially added to the DMF solution, and stirred at room temperature for 30 min. N-Boc-2-(2-Aminoethoxy) ethanamine (62 mg, 0.30 mmol) was added to the reaction system, and the mixture was stirred at room temperature for 12 h. The above-mentioned crude product was re-dissolved in DMSO (20 mL), and then TFA (1.5 mL) was added to the solution, followed by stirring at room temperature for 6 h. After removing the solvents in vacuo, the crude product of Azophenol-PEG_1_-NH_2_ was recrystallized in water and methanol to obtain 56 mg of yellow-brown powder with a yield of 69%.

IR-COOH (136 mg, 0.25 mmol) was dissolved in DMF (20 mL), EDC (78 mg, 0.50 mmol) and NHS (58 mg, 0.50 mmol) were sequentially added to the DMF solution, and stirred at room temperature for 30 min. Azophenol-PEG_1_-NH_2_ (78 mg, 0.23 mmol) was added to the reaction system, and the mixture was stirred at room temperature for 12 h. After removing the solvents *in vacuo*, the crude product was recrystallized in water and methanol to obtain 157 mg with a yield of 80%.^1^H NMR (400 MHz, DMSO-*d*_6_) δ: 10.39 (s,1H), 8.63 (t, *J* = 2.8 Hz, 1H), 8.26 (dd, *J* = 7.1, 5.0 Hz, 2H), 8.02 (d, *J* = 4.2 Hz, 2H), 7.85 (dd, *J* = 7.7, 4.3 Hz, 4H), 7.65 – 7.61 (m, 2H), 7.46 – 7.42 (m, 4H), 7.34 – 7.28 (m, 2H), 6.98 – 6.93 (m, 2H), 6.77 (s, 2H), 6.37 (t, *J* = 7.2 Hz, 2H), 4.24 (q, *J* = 3.8 Hz, 2H), 3.54 (t, *J* = 3.0 Hz, 2H), 3.46 – 3.39 (m, 4H), 3.28 (s, 2H), 3.09 (q, *J* = 2.9 Hz, 2H), 2.74 – 2.70 (m, 4H), 1.89 – 1.83 (m, 2H), 1.69 – 1.66 (m, 12H), 1.31 (t, *J* = 3.6 Hz, 3H) ppm. ^13^C NMR (100 MHz, DMSO-*d*_6_) δ: 185.07, 175.86, 169.66, 162.79, 157.86, 150.36, 147.47, 144.81, 140.58, 137.36, 133.68, 129.96, 126.45, 122.19, 117.87, 112.17, 107.61, 105.86, 102.32, 92.75, 78.08, 69.86, 57.06, 54.16, 40.60, 40.18, 39.77, 30.35, 28.62, 27.78, 25.84, 25.70, 23.87, 11.56 ppm. Mass spectrum (ESI): [M]^+^: *m/z* calcd. for (C_51_H_56_ClN_6_O_4_^+^): 851.40461, found: 851.40511.

# 3. Supporting results and experimental raw data

**3.1 Structural and molecular weight characterization of compounds**

**Figure S1.** ^1^H NMR spectrum of C4A-NH_2_ (DMSO-*d*_6_, 400 MHz, 25 °C).

**Figure S2.** HR-ESI mass spectrum of C4A-NH_2_.

**Figure S3.** ^1^H NMR spectrum of ICG-COOH (DMSO-*d*_6_, 400 MHz, 25 °C).

**Figure S4.** HR-ESI mass spectrum of ICG-COOH.

**Figure S5.** ^1^H NMR spectrum of ICG-C4A (DMSO-*d*_6_, 400 MHz, 25 °C).

**Figure S6.** ^13^C NMR spectrum of ICG-C4A (DMSO-*d*_6_, 100 MHz, 25 °C).

**Figure S7.** HR-ESI mass spectrum of ICG-C4A.

**Figure S8.** ^1^H NMR spectrum of IR-COOH (DMSO-*d*_6_, 400 MHz, 25 °C).

**Figure S9.** HR-ESI mass spectrum of IR-COOH.

**Figure S10.** ^1^H NMR spectrum of IR-C4A (DMSO-*d*_6_, 400 MHz, 25 °C).

**Figure S11.** ^13^C NMR spectrum of IR-C4A (DMSO-*d*_6_, 100 MHz, 25 °C).

**Figure S12.** HR-ESI mass spectrum of IR-C4A.

**Figure S13.** ^1^H NMR spectrum of Azophenol-PEG_1_-NH_2_ (DMSO-*d*_6_, 400 MHz, 25 °C).

**Figure S14.** ^1^H NMR spectrum of ICGA (DMSO-*d*_6_, 400 MHz, 25 °C).

**Figure S15.** ^13^C NMR spectrum of ICGA (DMSO-*d*_6_, 100 MHz, 25 °C).

**Figure S16.** HR-ESI mass spectrum of ICGA.

**Figure S17.** ^1^H NMR spectrum of IRA (DMSO-*d*_6_, 400 MHz, 25 °C).

**Figure S18.** ^13^C NMR spectrum of IRA (DMSO-*d*_6_, 100 MHz, 25 °C).

**Figure S19.** HR-ESI mass spectrum of IRA.

## 3.2 Collision cross section (CCS)

**Table S1.** Collision cross section (CCS) of self-inclusion and binary inclusion

| CCS(Å^2^) | 10.0 (μM) | 1.0 (μM) | 0.1 (μM) |
| --- | --- | --- | --- |
| ICGC@C4A | 335.72 | — | — |
| ICG-C4A | 355.59 | 356.58 | 355.39 |
| IRC@C4A | 336.64 | — | — |
| IR-C4A | 361.19 | 361.58 | 362.07 |

**FigureS20**. Two-dimensional ion mobility-mass spectrometry (IM-MS) contour plots of (a) ICGC@C4A (10.0 µM), (b) ICG-C4A (10.0 µM), (c) ICG-C4A (1.0 µM), and (d) ICG-C4A (0.1 µM), displaying the correlation between drift time (ms) and observed mass-to-charge ratio (m/z). The color gradient represents the signal intensity.

**FigureS21**. Two-dimensional ion mobility-mass spectrometry (IM-MS) contour plots of (a) IRC@C4A (10.0 µM), (b) IR-C4A (10.0 µM), (c) IR-C4A (1.0 µM), and (d) IR-C4A (0.1 µM), displaying the correlation between drift time (ms) and observed mass-to-charge ratio (m/z). The color gradient represents the signal intensity.

## 3.3 Binding affinities of self-inclusion

**Figure S22.** (a) Direct fluorescence titration of RhB (0.50 μM) with C4A (up to 9.81 μM) in PBS (10 mM, pH = 7.4) at 25 °C, *λ*_ex_ = 554 nm; (b) The associated titration curve at *λ*_em_ = 578 nm was fitted according to a 1:1 binding stoichiometry. (c) Competitive fluorescence titration of RhB@C4A (0.50/0.50 μM) with ICGC (up to 4.67 μM) in PBS (10 mM, pH = 7.4) at 25 °C, *λ*_ex_= 554 nm; (d) The associated titration curve at *λ*_em_ = 578 nm was fitted according to a 1:1 binding stoichiometry. (e) Competitive fluorescence titration of RhB@C4A (0.50/0.50 μM) with IRC (up to 3.73 μM) in PBS (10 mM, pH = 7.4) at 25 °C, *λ*_ex_= 554 nm; (f) The associated titration curve at *λ*_em_ = 578 nm was fitted according to a 1:1 binding stoichiometry. (g) Direct fluorescence titration of SiPcN_2_ (0.50 μM) with C4A (up to 2.15 μM) in PBS (10 mM, pH = 7.4) at 25 °C, *λ*_ex_ = 660 nm; (h) The associated titration curve at *λ*_em_ = 691 nm was fitted according to a 1:1 binding stoichiometry. (i) Direct fluorescence titration of SiPcN_2_ (0.50 μM) with ICG-C4A (up to 0.48 mM) in PBS (10 mM, pH = 7.4) at 25 °C, *λ*_ex_ = 660 nm; (j) The associated titration curve at *λ*_em_ = 691 nm was fitted according to a 1:1 binding stoichiometry. (k) Direct fluorescence titration of SiPcN_2_ (0.50 μM) with IR-C4A (up to 0.54 mM) in PBS (10 mM, pH = 7.4) at 25 °C, *λ*_ex_ = 660 nm; (l) The associated titration curve at *λ*_em_ = 691 nm was fitted according to a 1:1 binding stoichiometry.

## 3.4 UV-Vis absorption of dyes

**Figure S23.** (a) UV-Vis absorption spectroscopy of different concentrations of ICGC in PBS solution. (b) Standard curve of ICGC absorption at 776 nm as a function of concentration. (c) UV-Vis absorption spectroscopy of different concentrations of IRC in PBS solution. (d) Standard curve of IRC absorption at 770 nm as a function of concentration. (e) UV-Vis absorption spectroscopy of different concentrations of ICG-C4A in PBS solution. (f) Standard curve of ICG-C4A absorption at 724 nm as a function of concentration. (g) UV-Vis absorption spectroscopy of different concentrations of IR-C4A in PBS solution. (h) Standard curve of IR-C4A absorption at 718 nm as a function of concentration. (i) UV-Vis absorption spectroscopy of different concentrations of ICGA in PBS solution. (j) Standard curve of ICGA absorption at 735 nm as a function of concentration. (k) UV-Vis absorption spectroscopy of different concentrations of IRA in PBS solution. (l) Standard curve of IRA absorption at 726 nm as a function of concentration. (m) UV-Vis absorption spectroscopy of different concentrations of ICGC@C4A in PBS solution. (n) Standard curve of ICGC@C4A absorption at 735 nm as a function of concentration. (o) UV-Vis absorption spectroscopy of different concentrations of IRC@C4A in PBS solution. (p) Standard curve of IRC@C4A absorption at 730 nm as a function of concentration.

**FigureS24.** The UV–Vis absorption spectra of ICGC recorded at different times in PBS solution (a), in 10% FBS (b), and under 808 nm laser irradiation (c). The UV–Vis absorption spectra of ICGA recorded at different times in PBS solution (d), in 10% FBS (e), and under 808 nm laser irradiation (f). The UV–Vis absorption spectra of ICGC@C4A recorded at different times in PBS solution (g), in 10% FBS (h), and under 808 nm laser irradiation (i). The UV–Vis absorption spectra of ICG-C4A recorded at different times in PBS solution (j), in 10% FBS (k), and under 808 nm laser irradiation (l).

**FigureS25.** The UV–Vis absorption spectra of IRC recorded at different times in PBS solution (a), in 10% FBS (b), and under 808 nm laser irradiation (c). The UV–Vis absorption spectra of IRA recorded at different times in PBS solution (d), in 10% FBS (e), and under 808 nm laser irradiation (f). The UV–Vis absorption spectra of IRC@C4A recorded at different times in PBS solution (g), in 10% FBS (h), and under 808 nm laser irradiation (i). The UV–Vis absorption spectra of IR-C4A recorded at different times in PBS solution (j), in 10% FBS (k), and under 808 nm laser irradiation (l).

## 3.5 Hypoxia-responsive behaviors

**Figure S26.** (a) Fluorescence spectra of ICGC at various concentrations in PBS solution, *λ*_ex_ = 776 nm. (b) Calibration curve of fluorescence intensity at 805 nm as a function of ICGC concentration. (c) Fluorescence spectra of IRC at various concentrations in PBS solution, *λ*_ex_ = 770 nm. (d) Calibration curve of fluorescence intensity at 790 nm as a function of IRC concentration. Fluorescence spectra of ICG-C4A (1.0 μM) recorded at different times under normoxic (e) and hypoxic (f) conditions. Fluorescence spectra of IR-C4A (1.0 μM) recorded at different times under normoxic (g) and hypoxic (h) conditions.

**Table S2**. Photophysical properties of ICGC and IRC

| Name | Abs._Max_ (nm) | Em._Max_ (nm) | ε (M^−1^cm^−1^) | Q.Y. |
| --- | --- | --- | --- | --- |
| ICGC | 776 | 805 | 69090 | 0.012 |
| IRC | 770 | 790 | 86860 | 0.013 |
| ICGC-NH_2_ | 775 | 805 | 68700 | 0.010 |
| IRC-NH_2_ | 773 | 791 | 84220 | 0.012 |

ICGC-NH₂ and IRC-NH₂ are the arylamine-containing derivatives of ICGC and IRC, respectively, generated from ICG-C4A and IR-C4A upon hypoxic activation. Experimental conditions: PBS solutions, pH 7.4, 25 °C.

**FigureS27**. (a) Extracted ion chromatogram (XIC) of NH₂C4A, precursor ion [M+H]^+^ at m/z 485.22, retention time = 3.67 min. (b) MS/MS product-ion mass spectrum of NH₂C4A (precursor ion m/z 485.22). (c) Extracted ion chromatogram (XIC) of ICGC-NH_2_, precursor ion M^+^ at m/z 772.42, retention time = 8.17 min. (b) MS/MS product-ion mass spectrum of ICGC-NH_2_ (precursor ion m/z 772.42).

**Figure S28.** (a) Fluorescence responses of ICG-C4A at 805 nm (*λ*_ex_ = 776 nm) to various reductants. The ICG-C4A (5.0 μM) was treated with microsomes (40.0 μg/mL), nicotinamide adenine dinucleotide phosphate (NADPH, 40.0 μM), nicotinamide adenine dinucleotide (NADH, 40.0 μM), homocysteine (Hcy, 40.0 μM), cysteine (Cys, 40.0 μM), glutathione (GSH, 40.0 μM) and no additive, along with a positive control containing both microsomes (40.0 μg/mL) and NADPH (40.0 μM) under normoxic or hypoxic condition. Incubation time is 30 minutes. (b) Fluorescence responses of IR-C4A at 790 nm (*λ*_ex_ = 770 nm) to various reductants. The IR-C4A (5.0 μM) was treated with microsomes (40.0 μg/mL), NADPH (40.0 μM), NADH (40.0 μM), homocysteine Hcy (40.0 μM), Cys (40.0 μM), GSH (40.0 μM) and no additive, along with a positive control containing both microsomes (40.0 μg/mL) and NADPH (40.0 μM) under normoxic or hypoxic condition. Incubation time is 30 minutes. All data are presented as means ± SD, n = 3.

## 3.6 Pharmacokinetics

**FigureS29**. (a) Plasma concentrations of ICG-C4A and its control groups as a function of time following an intravenous (i.v.) injection. (Concentrations were determined based on the ICGC moiety for the ICGC and ICGC@C4A groups, and on the intact ICG-C4A conjugate for the ICG-C4A group) (b) Plasma concentrations of IR-C4A and its control groups as a function of time following an intravenous (i.v.) injection. (Concentrations were determined based on the IRC moiety for the IRC and IRC@C4A groups, and on the intact IR-C4A conjugate for the IR-C4A group) All data are presented as means ± SD, n = 3.

**Table S3**. Pharmacokinetic parameters of ICGC, ICGC@C4A, ICG-C4A, IRC, IRC@C4A and IR-C4A. *c*_max_: maximum plasma concentration; *t*_eli_: half-life of drug in plasma; AUC: area under curve of plasma concentration as a function of time; Vd: volume of distribution; CL: total plasma clearance.

| Formulations | *c*_max_ (μM) | *t*_eli_ (h) | AUC_0−∞_ (μM h) | V*_d_* (L kg^-1^) | CL (L kg^-1^ h^-1^) |
| --- | --- | --- | --- | --- | --- |
| ICGC | 1.45 ± 0.20 | 2.4 ± 0.1 | 0.87 ± 0.11 | 6.7 ± 0.8 | 1.90 ± 0.37 |
| ICGC@C4A | 1.53 ± 0.36 | 4.2 ± 0.2 | 2.15 ± 0.34 | 4.6 ± 0.5 | 0.77 ± 0.12 |
| ICG-C4A | 1.67 ± 0.15 | 6.1 ± 0.5 | 4.65 ± 0.72 | 3.1 ± 0.5 | 0.36 ± 0.13 |
| IRC | 1.74 ± 0.27 | 4.4 ± 0.3 | 1.42 ± 0.36 | 7.8 ± 0.4 | 1.22 ± 0.21 |
| IRC@C4A | 1.88 ± 0.41 | 5.3 ± 0.2 | 2.42 ± 0.55 | 5.9 ± 0.6 | 0.72 ± 0.28 |
| IR-C4A | 1.94 ± 0.38 | 7.0 ± 0.6 | 4.43 ± 0.87 | 3.9 ± 0.3 | 0.39 ± 0.10 |

## 3.7 *Ex vivo* fluorescence imaging

**Figure S30.** (a) *Ex vivo* fluorescence images of dissected major organs (heart, liver, spleen, lung and kidney) and tumor after injection of ICGC, ICGC@C4A and ICG-C4A at 48 h. (b) *Ex vivo* fluorescence images of dissected major organs (heart, liver, spleen, lung and kidney) and tumor after injection of IRC, IRC@C4A and IR-C4A at 48 h.

## 3.8 *In vivo* safety evaluation

**Figure S31.** **(a)** Cell populations of WBC, LYMPH and Gran were assayed for each mouse from different groups. Data are presented as they are. **(b)** Cell populations of PLT and RBC were assayed for each mouse from different groups. Data are presented as they are. **(c−g)** Plasma ALT (c), AST (d), ALP (e), BUN (f) and CREA (g) levels of each mouse in different groups at the end of the evaluation period. All data represent the mean ± s.d. (*n* = 3) and statistical significance (to the Saline group) was assessed by one-way ANOVA with Tukey’s test. n.s. for non-significant.

**Figure S32.** H&E staining sections of major viscera of mice in Saline, ICGC, ICGC@C4A, and ICG-C4A. The scale bar is 100 µm.

**Figure S33.** **(a)** Cell populations of WBC, LYMPH and Gran were assayed for each mouse from different groups. Data are presented as they are. **(b)** Cell populations of PLT and RBC were assayed for each mouse from different groups. Data are presented as they are. **(c−g)** Plasma ALT (c), AST (d), ALP (e), BUN (f) and CREA (g) levels of each mouse in different groups at the end of the evaluation period. All data represent the mean ± s.d. (*n* = 3) and statistical significance (to the Saline group) was assessed by one-way ANOVA with Tukey’s test. n.s. for non-significant.

**Figure S34.** H&E staining sections of major viscera of mice in Saline, IRC, IRC@C4A, and IR-C4A. The scale bar is 100 µm.

## References

[1]	W. M. Nau, X. Zhang, *J. Am. Chem. Soc.* **1999**, 121, 8022-8032.

[2]	S.-Y. Yao, K. Cai, D.-S. Guo, *Acta Polym. Sin.* **2022**, 53, 1271-1278.

[3]	S. G. König, S. Öza, R. Krämer, *Chem. Commun.* **2015**, 51, 7360-7363.

[4]	M. Gao, X. Huang, Z. Wu, Li. Wang, S. Yuan, Z. Du, S. Luo, R. Li, W. Wang, *Mater. Today Bio* **2022**, 15, 100316.
